# Supplementary material for: Transgene removal using an in cis programmed homing endonuclease via single-strand annealing in the mosquito Aedes aegypti
Source: Commun Biol. 2024 May 29;7:660. doi: 10.1038/s42003-024-06348-6 (PMC11137009; doi:10.1038/s42003-024-06348-6)
Supplement: Supplementary file 2 — Supplementary Information [file 42003_2024_6348_MOESM2_ESM.pdf]

Supplementary Information

Transgene removal using an *in cis* programmed homing endonuclease via single-strand annealing in the mosquito *Aedes aegypti*

Keun Chae, Bryan Contreras, Joseph S. Romanowski, Chanell Dawson, Kevin M. Myles, and Zach N. Adelman*

Affiliation: Department of Entomology, Texas A&M University, College Station, TX 77843, USA

***Correspondence:** Zach N. Adelman (zachadel@tamu.edu)

Supplementary Note 1

Analyzing 'amplicon_sorter' groups and unaligned reads directly

'Amplicon_sorter' filtered raw .fastq reads by size (0.5-kb) and binned reads into groups based on sequence similarity - referred to as 'consensus groups' in the paper. The purpose of read binning and creation of 'consensus groups' was to detect all editing outcomes regardless of alignment. Reads were binned into consensus groups if they were 80% similar, if not they were binned into unique groups. In total, 4 'consensus groups' were formed (n= 1361/1783 total reads) and 1 'unique' group was formed that did not meet the 80% cutoff criteria (n= 422/1783 total reads). These reads were retained as .fastq files and aligned using minimap2 to exclude secondary alignments and alignments with mapping qualities less than 20 in order to increase confidence in alignments (note: this decreased initial total alignments reported

from 991/1783 to 794/1783 total reads). Thus, with our new alignment parameters, 989/1783 reads did not align to signature sequences, but met the 0.5-kb cutoff filter.

Further interrogation of reads contained within the ‘unique’ groups from ‘amplicon_sorter’ were aligned using Seqman Ultra to the entire *P5* transgene and revealed 1,008 reads contained that did not contain the primer landing sites, but spanned the DsRED open reading frame and SV40 sequence part of the intended amplicon. It is possible these reads could be error-prone PCR amplicons or incomplete linear nanopore reads, in which case even small amounts of mismatches or shorter truncations present in raw reads may result in unsuccessful alignment to the 7 signature sequences. Further, to bypass potential errors from ‘amplicon_sorter’, we analyzed unaligned reads contained within the .sam file output from minimap2, used ‘fastq-filter’ to isolate 0.5-kb or larger reads (1783 total reads remained), and performed a new alignment using the entire *P5* transgene as a reference and minimap2 under higher alignment sensitivity parameters (namely, by decreasing the minimizer k-mer seed length from 15 to 5 to allow more alignments with shorter exact matches). After visualizing on IGV, 109 aligned reads spanning the DsRED open reading frame and SV40 sequence were recovered. Though fewer reads were aligned likely due to the alignment stringency of minimap2 even with manual changes to the alignments parameters, the consistency in the mapping location of these alignments by minimap2 and Seqman Ultra adds confidence that these reads are either incomplete or of lower quality, and as a result were either unaligned or filtered out during fastq quality control steps.

Increasing alignment sensitivity to catch unaligned reads

Next, sensitivity of minimap2 alignment was increased by decreasing the minimizer k-mer seed length from 15 to 10 to catch previously unaligned raw reads - these changes in alignment parameters decreased the minimum number of exact matches required for alignment initialization and allowed for less-specific alignments. Total aligned reads only modestly increased from 794 to 831 and SSA repair product frequencies remained nearly identical (59.32% by SV40, 34.06% by I-CmoeI, 3.61% by I-AniI, 3.01 %

by loxP, 0% by I-SceI, 0% by I-PpoI, and 0% by I-CreI). To further increase sensitivity, in addition to decreased minimizer k-mer seed length, previous mapping quality score filters were removed entirely. As a result, 1294 total reads aligned. Noticeable decreases in alignment specificity were observed by an increase in overall supplementary alignments and shorter/truncated alignments. Importantly, the increase in overall alignments did not change the relative frequency of SSA repair events (49% by SV40, 41% by I-CmoeI, 3.17% by I-AniI, 2.93% by loxP, 2.32% by I-SceI, 0.62% by I-PpoI, and 0.23% by I-CreI). Taken together, these unaligned reads can be recovered, but seemingly at the expense of alignment specificity.

Supplemental Note 2

The following DNA templates were considered diagnostic for SSA occuring between the indicated direct repeat sequence in the polyUb #P5 transgene.

SV40_101, gaattcGGCCGGCCTAGGcgcgccactagtgatatctactcgagtgaattcttatttgaggaggtttctctgtaaataatgatcgtagcagctcacggtta; I-AniI_67, tagggataacagggtaattatttgaggaggtttctctgtaaataatgatcgtagcagctcacggtta; I-CmoeI_96, AAAGCTTATCGATAcgcgtataacttcgtatagcatacattatacgaagttatatcgtagcagctcacggttacaaaacgtcgtgagacagtttgg; loxP_103, AAAGCTTATCGATAcgcgtataacttcgtatagcatacattatacgaagttatgtagtgccccaactggggtaacctttgagttctctcagttgggggcgtag, I-CreI_76, ttatttgaggaggtttctctgtaaataatgactctcttaaggtagcctagaacaaaacgtcgtgagacagtttggt; I-PpoI_69, tagggataacagggtaattatttgaggaggtttctctgtaaataatgactctcttaaggtagcctagaa; I-SceI_60, atcgtagcagctcacggttagggataacagggtaatgactctcttaaggtagcctagaaa

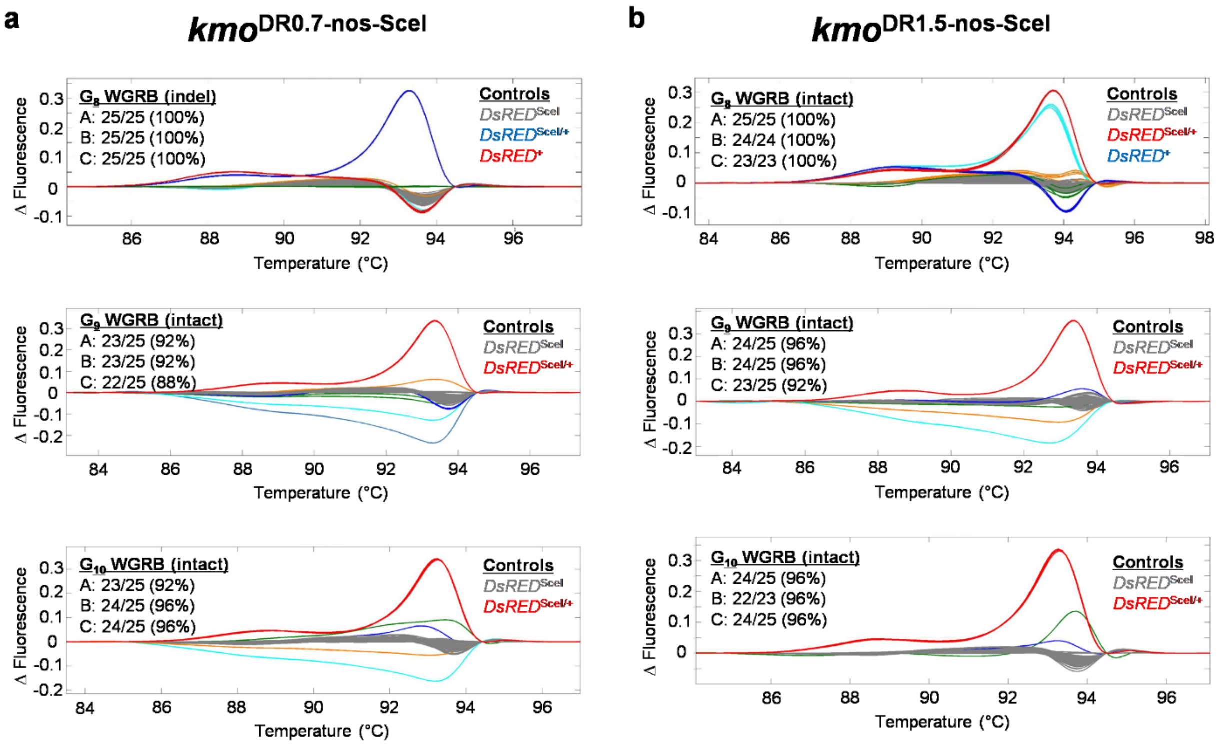

Supplemental Fig. S1 HRMA-based genotyping analysis for the multi-generational test of 1 piece-SSA strains. In DNA repair-dependent phenotype screening, WGRB females (*n* = 25 per replicate) of *kmo*$^{\text{DR0.7-nos-SceI}}$ (**a**) and *kmo*$^{\text{DR1.5-nos-SceI}}$ (**b**) were genotyped by HRMA using primers Hsp70-F and DsRED-5Ra to remove mosquitoes with a silent indel mutation occurred at *DsRED* from the mother groups for the next generation. A plasmid containing *DsRED* engineered with the I-*Sce*I site (*DsRED*^{SceI}) or no modification (*DsRED*$^{+}$) was utilized for the differential melting curve pattern control. The 1:1 mixture of the two plasmids was the heterozygous control (*DsRED*$^{\text{SceI/+}}$).

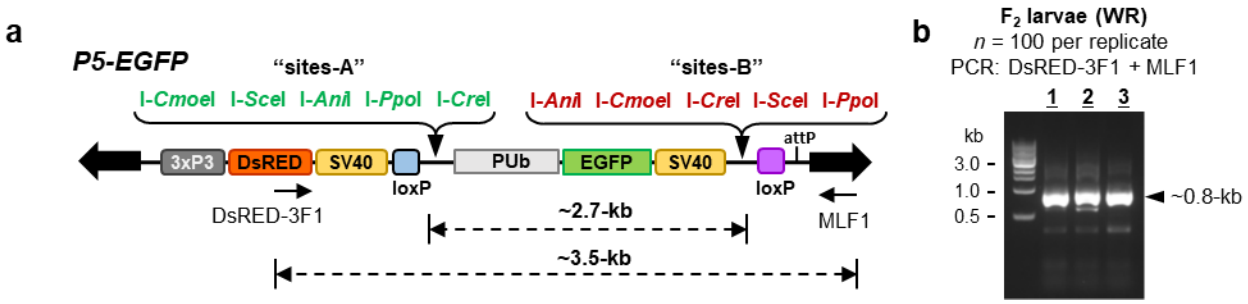

Supplemental Fig. S2 Amplification of *P5-EGFP* transgene from phenotypic WR individuals. **a** Schematic representation of *P5-EGFP* and two PCR primer sites, DsRED-3F1 and MLF1. **b** PCR amplicons obtained from genomic DNAs of F_2 mosquitoes scored as WR (white and $DsRED^+$ eyes), are substantially truncated, consistent with complete removal of the ~2.7-kb PUb-EGFP-SV40 cassette from the transgenic region.

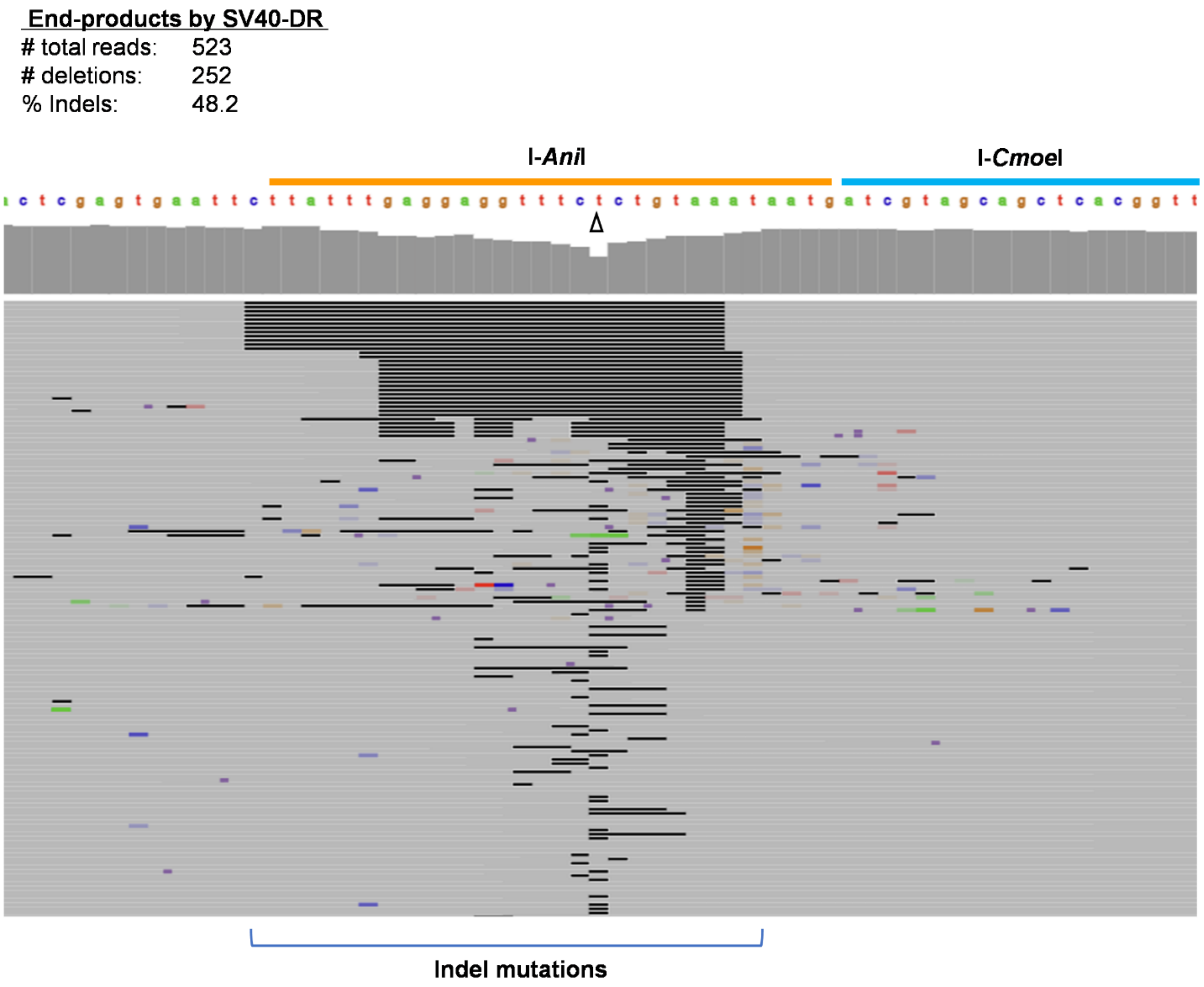

Supplemental Fig. S3 The accumulation of various indels at the I-*Ani*I sequence, which was maintained intact after the SSA-based DSB repair process mediated by SV40-DR. Among total crude reads (*n* = 523) of SV40-based SSA end products, about half (*n* = 252) showed to have the deletion of a nucleotide (arrowhead) that is a protruding end of I-*Ani*I-processed DNAs.

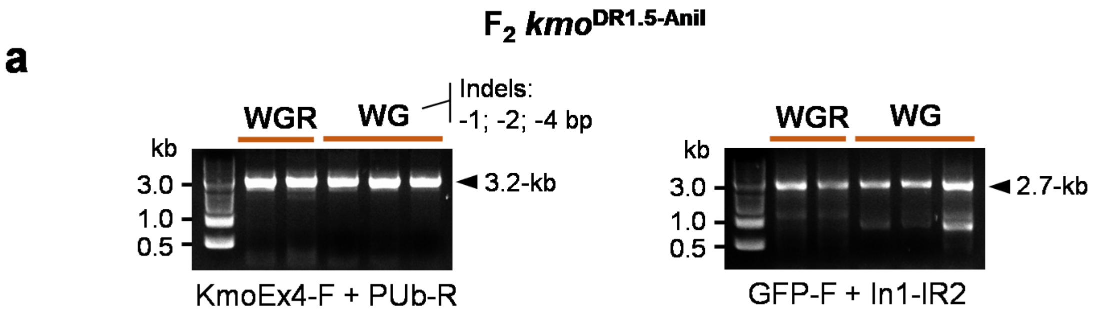

b

F_2 *kmo* $^{DR1.5-AniI}$	*I-AniI*	Indels
**WGR**	GCCACC**ATG**TATTTGAGGAGGTTTCTCTGTAAATACGTGCGCTCC	**0**
**WG**	GCCACC**ATG**TATTTGAGGAGGTTTCT----AAATACGTGCGCTCC	**-4**
**WG**	GCCACC**ATG**TATTTGAGGAG-TTTCTCTGTAAATACGTGCGCTCC	**-1**
**WG**	GCCACC**ATG**TATTTGAGGAGGTTTCTGT--AAATACGTGCGCTCC	**-2**

Supplemental Fig. S4 The NHEJ phenotype of F_2 *kmo*$^{DR1.5-AniI}$ was directly associated with indel mutations caused by the germline-specific activity of I-*Ani*I. a PCR analysis using a primer pair of KmoEx4-F and PUb-R verified the transgene integrity of WG (white eyes; $EGFP^+$ body) mosquitoes, identical to WGR (white and $DsRED^+$ eyes; $EGFP^+$ body). A primer pair of GFP-F and In1-IR2 were utilized to show their precise integration at the *kmo* locus. **b** Sequencing analysis revealed that indel mutations were generated at the I-*Ani*I sequence, which frameshifted the *DsRED* coding region.

Supplemental Figure 5. Uncropped images

Fig. 1b

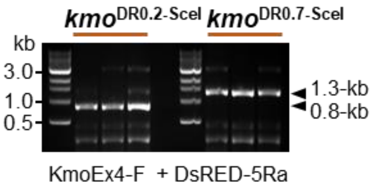

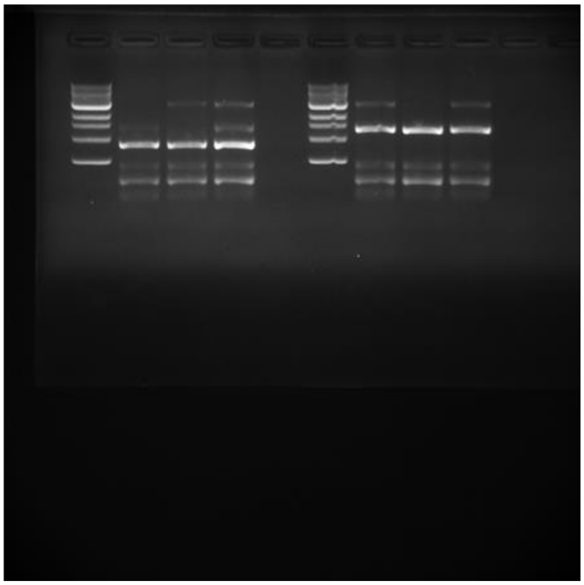

Fig. 2

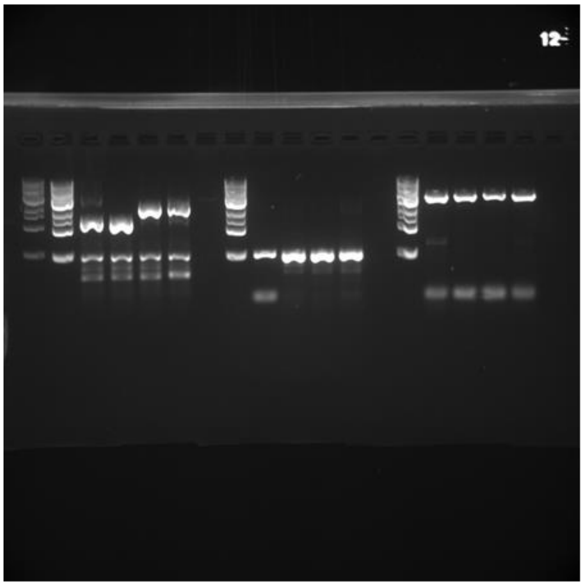

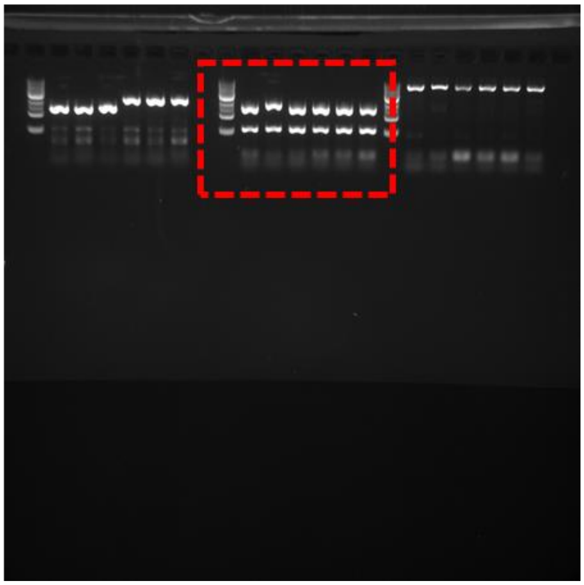

Fig. 5b

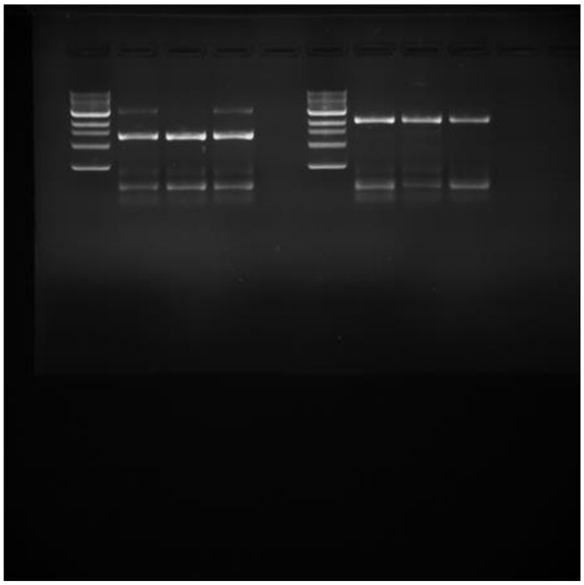

Supplemental Fig. S2

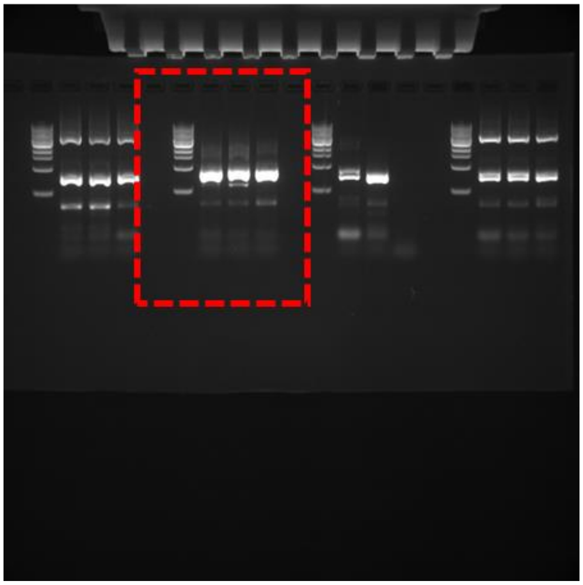

Supplemental Fig. S4a

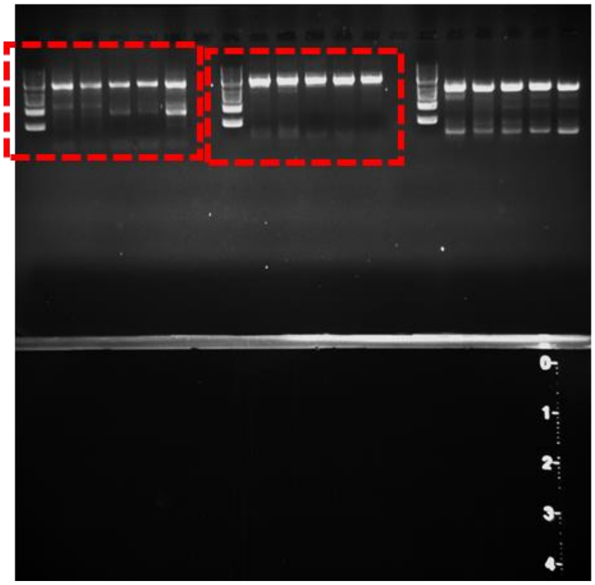

Supplemental Table S1. Generation of transgenic mosquito strains.

Transgenic mosquitoes	Donor DNAs	sgRNAs	Recipient strains	# Embryos injected	# G_0 Larvae survived	# G_0 Adults outcrossed w/ *kmo*$^{-/-}$	# G_1 Larvae w/ phenotypes				
							W	Blk	WG	WGR	WB
kmo$^{DR0.2\text{-}SceI}$	pSSA-KmoDR0.2-SceI	HybRED	*kmo*^{EGFP}	~2,000	314 (13.6%)	♂126 ♀142			22,337	**79** (0.4%)	
kmo$^{EGFP\#5}$	pBR-KmoEx4#5	KmoEx4	*Lvp*	~1,250	313 (25%)	♂140 ♀151	13,385		**68** (0.51%)		
kmo$^{DR1.5\text{-}AniI}$	pSSA-KmoDR1.5-AniI	HybRED	*kmo*^{EGFP}	~3,850	214 (5.6%)	♂83 ♀63			3,259	**13** (0.4%)	
nos-I-AniI	pMOS-3xP3-BFP-nos-AniI	—	*kmo*$^{-/-}$	~1,550	184 (11.8%)	♂74 ♀81	6,893				**5** (0.07%)
kmo$^{EGFP\#5\text{-}nos\text{-}SceI}$	pBR-KmoEx4#5-nos-SceI	KmoEx4	*Lvp*	~1,450	130 (9%)	♂57 ♀47		~20,000	**9** (0.05%)		
kmo$^{DR0.7\text{-}nos\text{-}SceI}$	pSSA-KmoDR0.7-nos-SceI	HybRED	*kmo*$^{EGFP\#5\text{-}nos\text{-}SceI}$	~2,500	195 (7.8%)	♂61 ♀65			4,546	**123** (2.7%)	
kmo$^{DR1.5\text{-}nos\text{-}SceI}$	pSSA-KmoDR1.5-nos-SceI	HybRED	*kmo*$^{EGFP\#5\text{-}nos\text{-}SceI}$	~2,500	321 (12.8%)	♂97 ♀102			5,420	**22** (0.4%)	

Marker phenotypes: W, white eyes; Blk, black eyes; G, EGFP; R, DsRED; B, BFP.

Supplemental Table S2. List of oligonucleotides for sgRNAs, PCR, and subcloning.

Oligonucleotides	Sequences (5' to 3')[a]
sgRNA-KmoEx4	GAAATTAATACGACTCACTATAGG**ATGAATGTTCGGGTACTTCT**GTTTTAGAGCTAGAAA
sgRNA-HybRED	GAAATTAATACGACTCACTATAGG**CGGTGCGGCCGCATAGGCGC**GTTTTAGAGCTAGAAA
KmoEx4-F	TGTGAGTAGATTCCTTCGTCGTTGG
KmoEx4-R	ATTGCGTAGCAAGTTTACCTTGGGC
DsRED-5Ra	TCACCTTCAGCTTCACGGTGTTGTGG
NosPro-3Fa	TGATCCTCGTTCGTCATAAAGG
SceI-5R	TCTTTCAGCAGTTTAGAGTTCGGACCCAGG
SV40-F	AATCAGCCATACCACATTTGTAGAGG
SV40-R	TTTTGCTCAGCGGCAAGATACATTGATGAGTTTGG
In1-IR2	AATCATGGGTAGGACGAATGTCTTACTCAGC
Hsp70-F	AGCAAAGTGAACACGTCGCTAAGCG
GFP-F	ATGGTGAGCAAGGGCGAGGAG
GFP-3R	CTTGTACAGCTCGTCCATGCCGAG
DSRED-3F1	TACCTGGTGGAGTTCAAGTCC
PUb-F	TTGGCACCAGCTGAGCCAGAC
PUb-R	TCCTGATTTGATCGACAATTTCGG
PUb-5R	CAATGCACAAGCTACATGTAAAG
MRF1	AAGACGATGAGTTCTACTGGCGTGGAATCC
MRR1	CTTGCCGTATGTGATGGAGCGTTGTCATGG
MLF1	TTGTTTACTCTCAGTGCAGTCAACATGTCG
MLR1	TTCGACAGTCAAGGTTGACACTTCACAAGG
KmoHA1-F-Kpn	GTCAGGTACCGCCAGATCGCAGATAGAGTGTGC
KmoDR0.2-R-Age	GTCAACCGGTAAGTAGCGCTGTTCTGGTGAACG
In1-IR2-AgAv	AAAAACCTAGGAAACCGGTAATCATGGGTAGGACGAATGTCTTACTCAGC
3'UTR#5-R-BmBp	TTTTTGCTCAGCGGATCCAGTCTGATTAATTAAGCAGGCC

a, sgRNA target sequences are shown in bold letters. Restriction enzyme site sequences were underlined.

Supplemental Table S3. The 1 piece-SSA test for transgene elimination of the *kmo*$^{DR\text{-}nos\text{-}SceI}$ strain at the G_8 generation.

G$_7$ Parental cross		G$_8$ Pupal screening										
♂	x ♀			total	WGRB		W		WGB		Blk	
				#	#	%	#	%	#	%	#	%
kmo$^{-/-}$	***kmo***$^{DR0.7\text{-}nos\text{-}SceI}$	♂	A:	413	200	48.4	209	50.6	1	0.49	3	1.47
			B:	330	157	47.6	167	50.6			6	3.68
			C:	283	141	49.8	142	50.2				
		♀	A:	504	227	45	271	53.8			6	2.58
			B:	399	204	51.1	190	47.6	2	0.96	3	1.44
			C:	281	130	46.3	150	53.4	1	0.76		
kmo$^{-/-}$	***kmo***$^{DR1.5\text{-}nos\text{-}SceI}$	♂	A:	229	102	44.5	120	52.4	4	3.67	3	2.75
			B:	68	41	60.3	27	39.7				
			C:	279	143	51.3	134	48	2	1.38		
		♀	A:	281	131	46.6	144	51.2	4	2.92	2	1.46
			B:	96	53	55.2	43	44.8				
			C:	391	197	50.4	188	48.1	1	0.49	5	2.46

Marker phenotypes: W, white eyes; Blk, black eyes; G, EGFP; R, DsRED; B, BFP.

Supplemental Table S4. The 1 piece-SSA test for transgene elimination of the *kmo*$^{DR\text{-}nos\text{-}SceI}$ strain at the G_9 generation.

G_8 Parental cross ♂	x ♀			G_9 Pupal screening: total #	WGRB #	WGRB %	W #	W %	WGB #	WGB %	Blk #	Blk %
kmo$^{-/-}$	***kmo***$^{DR0.7\text{-}nos\text{-}SceI}$	♂	A:	760	324	42.6	405	53.3	20	5.63	11	3.1
			B:	731	346	47.3	358	49	18	4.83	9	2.41
			C:	541	275	50.8	266	49.2				
		♀	A:	686	322	46.9	337	49.1	17	4.87	10	2.87
			B:	495	244	49.3	237	47.9	6	2.33	8	3.1
			C:	525	268	51.1	256	48.8			1	0.37
kmo$^{-/-}$	***kmo***$^{DR1.5\text{-}nos\text{-}SceI}$	♂	A:	544	262	48.2	276	50.7	3	1.12	3	1.12
			B:	287	142	49.5	145	50.5				
			C:	630	276	43.8	347	55.1			7	2.47
		♀	A:	490	228	46.5	257	52.5	3	1.29	2	0.86
			B:	370	187	50.5	183	49.5				
			C:	529	271	51.2	248	46.9	2	0.71	8	2.85

Marker phenotypes: W, white eyes; Blk, black eyes; G, EGFP; R, DsRED; B, BFP.

Supplemental Table S5. The 1 piece-SSA test for transgene elimination of the ***kmo***$^{DR\text{-}nos\text{-}SceI}$ **strain at the** G_{10} **generation.**

G_9 Parental cross		G_{10} Pupal screening										
♂	x ♀			total	WGRB		W		WGB		Blk	
				#	#	%	#	%	#	%	#	%
kmo$^{-/-}$	***kmo***$^{DR0.7\text{-}nos\text{-}SceI}$	♂	A:	803	201	25	565	70.4	17	7.14	20	8.4
			B:	600	144	24	440	73.3	8	5	8	5
			C:	658	197	29.9	461	70.1				
		♀	A:	634	162	25.6	437	68.9	15	7.61	20	10.2
			B:	320	103	32.2	207	64.7	6	5.31	4	3.54
			C:	642	167	26	475	74				
kmo$^{-/-}$	***kmo***$^{DR1.5\text{-}nos\text{-}SceI}$	♂	A:	585	130	22.2	446	76.2	6	4.32	3	2.16
			B:	688	157	22.8	530	77			1	0.63
			C:	689	193	28	465	67.5	15	6.7	16	7.14
		♀	A:	340	90	26.5	246	72.4	1	1.06	3	3.19
			B:	616	133	21.6	483	78.4				
			C:	599	160	26.7	425	71	8	4.6	6	3.45

Marker phenotypes: W, white eyes; Blk, black eyes; G, EGFP; R, DsRED; B, BFP.

Supplemental Table S6. Inverse PCR analysis for chromosomal sequences flanking the transgene in the *Nos-I-AniI* strain.

Mariner Mos1 transgenic lines	The targeted gene	Flanking Sequences
nos-I-AniI	AAEL010783<br>(Chromsome2)	AATTTCAGATAGCAATATTTCAATATTTATGGACTATCCTACTTC<br>GCTGGAAAATTTATGTAATCAGTTTGAGTCAGCGATGTGTTTTGT<br>GAGCATCGAGAGGTTTGATGAGATTGTGAGGGCTGTTCGGAAAAT<br>GTGTGAGAGAGCGAGAATGAGGTATTCGGGAGCATCAAGAGGATC<br>TTTCAAGGATA-**RR-MOS-nos-AniI-LR**-TACACATGGGGTGG<br>GAGAGAGAATGTCAGAAATCAACCCCCTTTTCAACGGAGGGTGGG<br>AAAAACAAGATGGCTGCTCTCCAGCGCTAGAAGGACAACTTGTCC<br>CCTAAGGATTCCCAGAGGCTCAGTTGAAGTGTTATAATGATTCAT<br>GGTACT

Supplemental Table S7. DNA repair events are induced on the transgene of the *P5-EGFP* strain by a homing endonuclease-expressing strain, *nos-I-AniI* or *nos-I-SceI*.

Parental cross (♂30 x ♀100)	Lineage of the nuclease	Replicates	F_2 Larval screening					
			# Total	# WGR **No DSB**; **NHEJ**	# WG **aberrant; NHEJ**	# WR **SSA; NHEJ**	% WR **SSA; NHEJ**	# W ***kmo***$^{-/-}$
nos-I-AniI x *P5-EGFP*	F_0♂-F_1♂	A	1974	929		46	4.72	999
		B	2300	1174	2	47	3.84	1077
		C	2049	1070	6	36	3.24	937
	F_0♂-F_1♀	A	1919	833	7	100	10.6	979
		B	1423	601	2	82	12	738
		C	895	457	1	30	6.15	407
	F_0♀-F_1♂	A	943	385	2	77	16.6	479
		B	2000	739	25	184	19.4	1052
		C	1484	583	9	185	23.8	707
	F_0♀-F_1♀	A	1454	495	2	224	31	733
		B	1750	697	9	146	17.1	898
		C	1891	740	19	166	17.9	966
nos-I-SceI x *P5-EGFP*	F_0♂-F_1♂	A	2364	1106		2	0.18	1256
		B	2220	1145		1	0.09	1074
		C	2167	1113				1054
	F_0♂-F_1♀	A	1090	535		1	0.19	554
		B	1748	903		1	0.11	844
		C	2096	1114		3	0.27	979
	F_0♀-F_1♂	A	1349	714	1	3	0.42	631
		B	2367	1243	1	1	0.08	1122
		C	2310	1084		6	0.55	1220
	F_0♀-F_1♀	A	1820	915		3	0.33	902
		B	1622	828		1	0.12	793
		C	1540	828		6	0.72	706

Marker phenotypes: W, white eyes; Blk, black eyes; G, EGFP; R, DsRED.
